# Adipose Mesenchymal Stem Cells Secreted Exosomal miR‐26a‐5p Ameliorate Renal Fibrosis via Inhibiting Glycolysis and Ferroptosis in Renal Interstitial Fibrosis

**DOI:** 10.1111/jcmm.71369

**Published:** 2026-10-16

**Authors:** Chi Zhang, Rui Ge, Min Liu, Jianhuai Huang, Jingyu Cai, Tao Sun, Sheng Ge, Qiuchen Li, Yuanyuan Zhang

**Affiliations:** ^1^ Department of Nephrology The Affiliated Suqian Hospital of Xuzhou Medical University Suqian Jiangsu China; ^2^ Jiangsu Key Laboratory of Immunity and Metabolism Xuzhou Medical University Xuzhou Jiangsu China; ^3^ Department of Nephrology Affiliated Hospital of Xuzhou Medical University Xuzhou Jiangsu China; ^4^ Department of Nephrology, Affiliated Hospital of Medical School, Suzhou Hospital Nanjing University Suqian Jiangsu China

**Keywords:** adipose mesenchymal stem cell, exosome, ferroptosis, microcirculation, renal interstitial fibrosis

## Abstract

Renal fibrosis, a defining feature of chronic kidney disease, involves a metabolic shift toward aerobic glycolysis and is frequently accompanied by ferroptosis, a form of iron‐dependent cell death. This study investigated the therapeutic potential of adipose mesenchymal stem cell‐derived exosomes (AMSC‐exo) in renal fibrosis. Using a unilateral ureteral obstruction (UUO) mouse model and hypoxia‐treated HK‐2 cells, we assessed fibrosis, glycolysis, mitochondrial morphology, and ferroptosis. miRNA sequencing, dual‐luciferase reporter assays, and transcriptome sequencing were performed to identify key exosomal miRNAs and downstream pathways. AMSC‐exo significantly attenuated UUO‐induced fibrosis and suppressed glycolysis and ferroptosis in tubular epithelial cells. miR‐26a‐5p was highly enriched in AMSC‐exo and downregulated 6‐phosphofructo‐2‐kinase/fructose‐2,6‐bisphosphatase 3 (Pfkfb3), a key regulator of glycolysis and ferroptosis. Knockdown of miR‐26a‐5p markedly weakened the renoprotective effects of AMSC‐exo, whereas Pfkfb3 inhibition reversed the effects induced by miR‐26a‐5p deficiency. These findings indicate that AMSC‐exo alleviate renal fibrosis by delivering miR‐26a‐5p to suppress Pfkfb3, thereby inhibiting glycolytic reprogramming and ferroptosis, and suggest the miR‐26a‐5p/Pfkfb3 axis as a potential therapeutic target for renal fibrosis.

**Abbreviations**
ACSL4: Acyl‐CoA synthetase long chain family member 4
AMSC‐exo: adipose mesenchymal stem cell‐derived exosome
CKD: chronic kidney disease
EdU: 5‐ethynyl‐2′‐deoxyuridine
FAO: fatty acid oxidation
GPX4: glutathione peroxidase 4
HIF‐1α: hypoxia‐inducible factor alpha
HK2: hexoskinase 2
HK‐2: human proximal tubular cells
LDHA: lactate dehydrogenase A
NC: negative control
NTA: nanoparticle tracking analysis
PBS: sterile phosphate buffer saline
Pfkfb3: 6‐phosphofructo‐2‐kinase/fructose‐2,6‐bisphosphatase 3
PFKM: phosphofructokinase, muscle type
ROS: reactive oxygen species
RT‐qPCR: quantitative real‐time PCR
TECs: tubular epithelial cells
TEM: transmission electron microscopy
TGF‐β1: transforming growth factor beta‐1
UUO: unilateral ureteral obstruction
α‐SMA: α‐smooth muscle actin

## 1 Introduction

Chronic kidney disease (CKD) inevitably advances to renal fibrosis, a persistent scarring process that deteriorates kidney function and culminates in end‐stage renal disease [1, 2]. This fibrosis entails the loss of TECs through cell death and dedifferentiation, marked by a decrease in epithelial markers and an increase in mesenchymal markers. Notably, elevated Notch, Wnt and Hedgehog signalling within TECs instigate this dedifferentiation, a characteristic feature of CKD [3]. Although TECs do not directly transform into myofibroblasts, they significantly contribute to fibrosis by promoting inflammation and collagen deposition. Transforming growth factor beta‐1 (TGF‐β1) plays a crucial role, propelling the production of fibrillary collagens and perpetuating harmful processes like cell death and dedifferentiation [4, 5]. Notably, current anti‐fibrotic therapies exhibit limited efficacy in reversing or halting this progression.

Therefore, exploring novel therapeutic strategies is essential. AMSCs have emerged as promising tools due to their diverse therapeutic properties, including promoting angiogenesis, immunomodulation, anti‐inflammation and supporting cell function [6, 7, 8]. Pre‐clinical research and clinical trials investigating the potential of MSCs have steadily increased over the past few decades [9]. Notably, these studies demonstrate the potential of AMSCs to enhance blood flow and kidney function and reduce inflammation in individuals with renovascular disease [10].

Exosomes, membrane‐bound vesicles secreted by various cells, are gaining recognition as vital messengers in intercellular communication [11]. Their unique properties, including low immunogenicity and biocompatibility, make them promising candidates for liquid biopsies and drug delivery systems, demonstrating success in treating diverse diseases [12]. Compared to cell‐based therapies, exosomes offer a safer and more targeted approach. Among various exosomes, those derived from MSCs have demonstrated particular promise in tissue repair, regeneration, and even inhibiting cancer progression [13, 14]. Their therapeutic potential stems from their unique cargo of bioactive molecules like nucleic acids, proteins and lipids, which can exert effects such as promoting angiogenesis and counteracting inflammation in recipient cells [11, 15].

TECs present an intriguing metabolic paradox. Despite heavily relying on mitochondrial fatty acid oxidation (FAO) for energy, they exhibit a unique response to transient hypoxia. Under such stress, FAO is significantly suppressed beyond the duration of the hypoxic insult, whereas carbohydrate oxidation fails to compensate [16, 17]. These metabolic constraints compel surviving TECs to undergo a phenotypic switch characterized by cytoskeletal rearrangements, increased extracellular matrix protein production, and a shift toward upregulated glycolysis [18]. This switch may also contribute to increased ferroptosis, a cell death pathway linked to iron dysregulation [19]. However, the precise role of AMSC‐exo in regulating TECs' metabolism and its impact on ferroptosis remains unclear.

This study aims to identify and quantify the content of AMSC‐exo and investigate the underlying pathways through which they modulate glycolysis and ferroptosis in a UUO mouse model, a well‐established model of renal fibrosis, as well as in hypoxia‐induced HK‐2 cells, serving as a cell model. By elucidating the mechanisms by which AMSC‐exo influences TECs' metabolism and ferroptosis, this study holds promise for developing novel therapeutic strategies to combat renal fibrosis and improve patient outcomes.

## 2 Methods

### 2.1 Exosome Isolation and Purification

AMSCs were procured using our previously described protocol and cultured in mesenchymal stem cell medium (MSCM; ScienCell, USA) supplemented with 5% foetal bovine serum (FBS; ScienCell) at 37°C and 5% CO_2_ for 48 h. The supernatants were collected and centrifuged at 2000 rpm for 20 min and then at 16,000 rpm for 30 min to remove impurities and cellular debris. The supernatant was ultracentrifuged at 180 min (Beckman Coulter Optima XPN‐100) at 4°C. The exosome pellets were resuspended in sterile phosphate buffer saline (PBS); for further purification, the pellets were passed through a 0.22‐μm filter and then stored at −80°C or used for ensuing experiments.

### 2.2 Exosome Characterization

The morphology of the isolated exosomes was characterized using transmission electron microscopy (TEM; HITACHI). To verify the particle number and size distribution of the exosomes, nanoparticle tracking analysis (NTA; Zetasizer version 6.12; Malvern Instruments, UK) was employed. Western blotting was then performed to analyse the expression of exosome‐associated protein markers, particularly CD81, CD9, and Tsg101. In addition, the negative marker Calnexin was assessed to evaluate potential contamination from intracellular organelles.

### 2.3 Animal Models and Therapeutic Experiments

Eight‐week‐old male C57BL/6 mice (21–24 g) were sourced from Nanjing Vital River Laboratory Animal Technology Co. Ltd. These mice were housed under controlled environmental conditions with a 50% ± 10% humidity, 24°C ± 1°C temperature, and a 12‐h light/dark cycle. To induce UUO and subsequent progressive renal interstitial fibrosis, a surgical procedure was performed. Mice were anaesthetised; a left abdominal incision was made; the left ureter was exposed and ligated with a 4‐0 silk suture. The incision was then closed. The mice were divided into three groups: sham‐operated (sham), UUO with PBS injection (UUO), and UUO with AMSC‐exo injection. AMSC‐exo was diluted in PBS to 1 × 10^3^ μg/mL. After 7 days, mice were sacrificed, and their kidneys were collected for further analysis, corresponding to the early stage of fibrogenic responses in the UUO model. To analyse the role of miR‐26a‐5p in exosome‐mediated kidney inflammation and angiogenesis, AMSCs were transfected with either miR‐26a‐5p inhibitors or negative control (NC) inhibitors. Subsequently, 100 μL of the secreted exosomes (miR‐26a‐5pIN‐exo or NC‐exo) were administered intravenously to UUO mice, along with the PBS control group. Animal experiments followed a pre‐approved protocol by the Institutional Animal Care and Use Committee (IACUC) of Xuzhou Medical University.

### 2.4 Cellular Culture and Treatment

Human proximal tubular epithelial cells (HK‐2), obtained from ScienCell Research Laboratories (USA), were cultured in Dulbecco's Modified Eagle Medium (DMEM; Bio‐Channel, China) supplemented with 10% FBS and 1% penicillin–streptomycin at 37°C in a humidified incubator with 5% CO_2_. To induce hypoxia, HK‐2 cells were exposed to 1% O_2_ in a hypoxic chamber for 24 h, while control cells were maintained under normoxic conditions (21% O_2_). All culture media used for hypoxic experiments were pre‐equilibrated under 1% O_2_ overnight prior to use to ensure stable oxygen levels.

For treatment experiments, HK‐2 cells under hypoxic conditions were incubated with AMSC‐exo at the indicated concentrations for 24 h.

For rescue experiments, cells were further treated with PFK15 (a Pfkfb3 inhibitor; MedChemExpress, Monmouth Junction, NJ, USA; Cat. No. HY‐100853) at a concentration of 10 μM for 24 h. PFK15 was dissolved in DMSO, and the final concentration of DMSO was kept consistent across all groups.

### 2.5 Transwell Migration Assay

The migratory ability of HK2 cells across various treatment groups was assessed using a transwell migration assay. In the upper chamber, 100 μL of cell suspension containing 5 × 10^5^ HK2 cells in FBS‐free DMEM was seeded. The lower chamber contained 500 μL of DMEM supplemented with 20% FBS as a chemoattractant. After 8 h (standard incubation period), the transwell inserts were extracted, washed twice with PBS, fixed with paraformaldehyde for 15 min, and washed again. Finally, the cells were stained with crystal violet solution for 20 min. The migrated cells were then quantified by counting five random microscopic fields.

### 2.6 HK2 Proliferation Assay

Based on the manufacturer's instructions, cell proliferation in the HK2 cultures was assessed using the 5‐ethynyl‐2′‐deoxyuridine (EdU) staining assay (Ribobio, Guangzhou, China). A total of 8 × 10^3^ HK2 cells were seeded per well in a 96‐well plate. Next, EdU labelling solution was added; the cells were incubated for 2 h. Subsequently, the cells were fixed with paraformaldehyde, washed with PBS, permeabilized with 0.5% Triton X‐100 for 20 min, and washed again with PBS. The cells were then incubated with 50 μL of click reaction solution for 30 min, followed by another PBS wash. Finally, the cells were stained with 50 μL of Hoechst 33342 for 10 min and visualized using an inverted fluorescence microscope (Leica, Germany).

### 2.7 AMSCs Exosome Transfection With miRNA Inhibitor

In vitro, AMSC‐exo was transfected with either the miR‐26a‐5p inhibitor or a NC inhibitor (GenePharma) at a concentration of 100 nM using Lipofectamine 3000 for 24 h, following the manufacturer's instructions. The sequences for the miR‐26a‐5p inhibitor and NC inhibitor are provided below:

hsa‐miR‐26a‐5p inhibitor: AGCCUAUCCUGGAUUACUUGAA.

miR‐NC inhibitor: CAGUACUUUUGUGUAGUACAA.

### 2.8 Histology and Immunofluorescence Staining

The extent of kidney injury was assessed using HE staining. To evaluate the level of tubulointerstitial fibrosis, Masson's trichrome staining and immunohistochemistry (IHC) for α‐smooth muscle actin (α‐SMA) were performed.

### 2.9 miRNA‐26a‐5p Analysis and RNA Sequencing

AMSSs‐exo‐extracted miRNA‐26a‐5p was sequenced by Shanghai Genechem Co. Ltd. to determine its precise sequence and abundance. Additionally, total RNA was extracted from the left kidney tissues of both UUO and sham groups for RNA sequencing analysis. This comprehensive approach will allow us to investigate the expression profile of miRNAs and mRNAs in the context of kidney injury.

### 2.10 Bioinformatic Analysis of miRNA Targets

To identify potential targets of miR‐26a‐5p, bioinformatic analysis was performed using three online databases: TargetScan (https://www.targetscan.org/), and miRWalk (http://mirwalk.umm.uni‐heidelberg.de/search_mirnas/).

### 2.11 Dual‐Luciferase Reporter Assay

To validate the interaction between miR‐26a‐5p and the predicted 6‐phosphofructo‐2‐kinase/fructose‐2,6‐bisphosphatase (Pfkfb3) 3′UTR binding site, a dual‐luciferase reporter assay was performed. Briefly, 293T cells were co‐transfected with either wild‐type Pfkfb3 3′UTR or mutant‐type Pfkfb3 3′UTR constructs and either miR‐26a‐5p mimics or inhibitors using Lipofectamine 3000 Reagent (Invitrogen, USA). Using the Dual‐luciferase Reporter Assay Kit (Promega, USA), luciferase activity was measured 48 h post‐transfection. The relative luciferase activity was determined by the ratio of Renilla luciferase activity to Firefly luciferase activity.

### 2.12 Lactate Assays

The lactate concentration of the cell supernatant and renal tissues was measured to analyse lactate production. All procedures were performed according to the manufacturer's instructions for the L‐Lactate Assay kit (Abcam, USA).

### 2.13 TEM Analysis for Mitochondria

To elucidate the ultrastructural characteristics of the left renal tissue, TEM analysis was conducted. The tissue underwent initial fixation in 2.5% glutaraldehyde (A17876; Alfa Aesar, USA) for 24 h at 4°C to conserve its intricate structural details. Post‐fixation, the tissue was subjected to dehydration via a series of ethanol solutions at increasing concentrations (30%, 50%, 70%, 90% and 100%) to eliminate water and prepare it for embedding. The dehydrated tissue was infiltrated with an epoxy‐acrylic resin blend under vacuum conditions to ensure thorough penetration. The resin blend was polymerized at 60°C for 48 h to form a rigid block. Ultrathin sections (~70–90 nm thick) were excised from the resin block using a glass knife on an ultramicrotome (Hitachi H‐7500, Japan), placed on copper grids, and stained with uranyl acetate and lead citrate to augment contrast and enhance cellular structure visualization. The stained sections were examined under a TEM (Hitachi H‐7500, Japan) at an accelerating voltage of 80 kV. Images were captured digitally and analysed using ImageJ software (https://imagej.nih.gov/ij/). The TEM analysis unveiled detailed morphological features of the renal tissue, including mitochondrial morphology, cristae structure, and evidence of mitochondrial damage within the cells.

### 2.14 Measurement of Mitochondrial Oxidation Levels by MitoSOX

Cells, seeded in six‐well plates, were exposed to various treatments as per the experimental design. Post‐treatment, cells were thoroughly washed with HBSS (Hanks' balanced salt solution; Wabcan, China) to eliminate residual media. Subsequently, each well was treated with 5 μM of the mitochondrial probe MitoSOX (Thermo Fisher Scientific, USA), and the plates were incubated for 30 min. Cells were then fixed with 4% paraformaldehyde for further analysis. Nuclei visualization was achieved through DAPI staining (Beijing Solarbio, China) for 10 min. Following three methanol washes for optimal image clarity, the plates were sealed. Mitochondrial oxidation levels were quantified by analysing MitoSOX fluorescence intensity using ImageJ software (https://imagej.net/ij/).

### 2.15 Mitochondrial Membrane Potential (MMP) Assay

MMP was evaluated using the JC‐1 fluorescent probe (C2006; Beyotime, China). HK2 cells were seeded in six‐well plates at a density of 10 × 10^4^ cells per well. Post‐treatment, as described in the “Cell Treatment” section, the cells were washed twice with PBS to remove residual treatment media. They were then incubated with 5 μM JC‐1 (Beyotime, China) for 20 min at 37°C in a dark environment. The cells were then washed thrice with JC‐1 staining buffer to remove any unbound dye. Finally, fluorescence intensity was analysed using an inverted fluorescence microscope (Leica, Germany). ImageJ software (https://imagej.nih.gov/ij/) was used to quantify the fluorescence intensity of each treatment group by measuring the ratio of red to green fluorescence.

### 2.16 RNA Isolation and Quantification

Total RNA was extracted from samples utilizing the RNAiso Plus reagent (Takara, Japan) in accordance with the manufacturer's guidelines. For mRNA quantification, reverse transcription was executed using the PrimeScript reverse transcription reagent (Takara, Japan). This enzyme converts single‐stranded mRNA into complementary DNA (cDNA), which can subsequently be amplified and quantified via real‐time PCR. The PrimeScript reagent, owing to its high efficiency and sensitivity, is optimal for mRNA quantification. The quantification of miRNA was performed using a real‐time PCR detection kit with miRNA‐specific primers (GeneCopoeia, USA). This kit employs a TaqMan probe‐based methodology, which involves a short, fluorescently labelled probe that anneals to the target miRNA sequence. All primers utilized in this study are enumerated in Table 1.

**TABLE 1:** Primers utilized for RT‐qPCR.

Genes	Forward primer	Reverse primer
TGF‐β (Homo)	GGAAATTGAGGGCTTTCGCC	CCGGTAGTGAACCCGTTGAT
Hif‐1α (Homo)	AAATGAGCTCCCAATGTCGG	CGACGTTCAGAACTTATCCTACCAT
Collagen (Homo)	CCCCGAGGCTCTGAAGGT	GCAATACCAGGAGCACCATTG
α‐SMA (Homo)	CCCCGGGAGCCAGTCT	GCTTCACAGGATTCCCGTCT
Pfkfb3 (Homo)	CCCTTCAGGAAAGCCTGTGG	GAACACTTTTGTGGGGACGC
Pfkfb3 (Mus)	CGGGAGAGGTCAGAGAACAT	CGATCACTTGGCCTCGAGAA
HK2 (Homo)	TCCCCTCTCGCGTCTCC	AGAGATACTGGTCAACCTTCTGC
HK2 (Mus)	GTTTCTCTATTTGGCCCCGAC	AGAGATACTGGTCAACCTTCTGC
PFKM (Homo)	AATCTGCAAGAAAGCAGCGG	GCAGCATTCATACCTTGGGC
PFKM (Mus)	GGTTTGGAAGCCTCTCCTCC	GGGTCATGATCCACTCTTGTAGT
LDHA (Homo)	TTGTCTCTGGCAAAGTGGAT	ACCGCTTCCAATAACACGGT
LDHA (Mus)	AGTAAGTCCTCAGGCGGCTA	GGACTTTGAATCTTTTGAGACCTTG
GPX4 (Homo)	GAAGATCCAACCCAAGGGCA	GACGGTGTCCAAACTTGGTG
GPX4 (Mus)	CCTCCCCAGTACTGCAACAG	GCACACGAAACCCCTGTACT
ACSL4 (Homo)	TTTTGCGAGCTTTCCGAGTG	AGCCGACAATAAAGTACGCAAAT
β‐actin (Homo)	ACAGAGCCTCGCCTTTGC	GATATCATCATCCATGGTGAGCTGG
β‐actin (Mus)	CACTGTCGAGTCGCGTCC	TCATCCATGGCGAACTGGTG
miR‐26b‐5p	CGCGTTCAAGTAATCCAGGA	AGTGCAGGGTCCGAGGTATT
U6	CTCGCTTCGGCAGCACAT	TTTGCGTGTCATCCTTGCG

### 2.17 Western Blotting Analysis

Cells and renal specimens from various treatment cohorts underwent lysis via RIPA buffer (Beyotime). Proteins were segregated using SDS‐PAGE on 8%–12% gels and subsequently transferred to PVDF membranes (Millipore, Jaffrey, NH, USA). These membranes were blocked with a 5% BSA solution for 1 h. Following this, they were incubated with primary antibodies: anti‐CD9 (20597‐1‐AP; Proteintech), anti‐CD81 (27855‐1‐AP; Proteintech), anti‐Tsg101 (28283‐1‐AP; Proteintech), anti‐Calnexin (10427‐2‐AP; Proteintech), anti‐HIF‐1α (20960‐1‐AP; Proteintech), anti‐α‐SMA (55135‐1‐AP; Proteintech), anti‐Collagen I (67288‐1‐Ig; Proteintech), anti‐TGF‐β1 (81746‐2‐RR; Proteintech), anti‐HK2 (22029‐1‐AP, Proteintech), anti‐PFKM (55028‐1‐AP; Proteintech), anti‐LDHA (21799‐1‐AP; Proteintech), anti‐PFKFB3 (13763‐1‐AP; Proteintech), anti‐GPX4 (67763‐1‐Ig; Proteintech), and anti‐ACSL4 (22401‐1‐AP; Proteintech). Post‐washing, the membranes were incubated with horseradish peroxidase (HRP)‐conjugated secondary antibodies, including β‐actin (AB2001; Abways) and GAPDH (AB2000; Abways).

### 2.18 Statistical Analysis

Statistical evaluations were performed utilizing the software Statistical Package for the Social Sciences (version 22.0). Data representation was in the form of mean ± standard error of the mean to depict both the central tendency and measurement variability. Independent samples *t*‐tests were employed for mean value comparisons between the two groups, with a significance level of *α* = 0.05 determining statistical significance.

## 3 Results

### 3.1 Characterization of AMSC‐Exo

TEM analysis authenticated the isolation of exosomes, exhibiting characteristic bilayer‐membraned vesicles within a size range of 50–200 nm (Figure 1A,B). The presence of exosomal markers (CD9, Tsg101 and CD81) was further substantiated by Western blotting. In contrast, the negative marker Calnexin was not detected in the exosome preparations, indicating minimal contamination from intracellular organelles (Figure 1C). RT‐qPCR demonstrated a significant upregulation of Hif‐1α, α‐SMA, collagen, and TGF‐β in HK2 cells under hypoxia (Figure 1D–G). Notably, this trend was reversed upon AMSC‐exo treatment, indicating their therapeutic potential against hypoxia‐induced renal fibrosis and inflammation.

**FIGURE 1:**
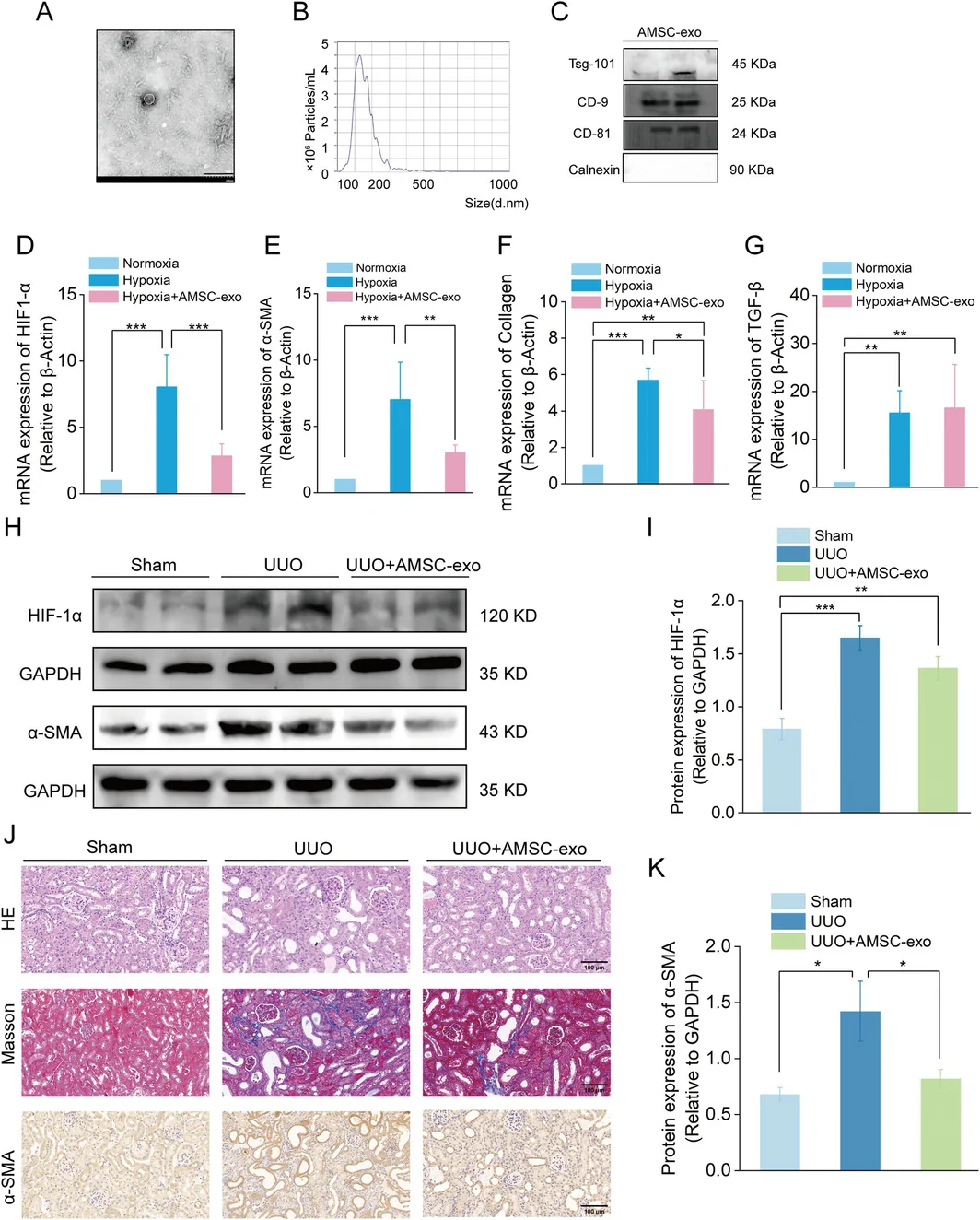
AMSC‐exo characterization and amelioration of UUO injury in mice. (A) TEM reveals AMSC‐exo morphology (scale bar: 200 nm). (B) Nanoparticle tracking analysis shows AMSC‐exo size distribution. (C) Western blot confirms the presence of exosome markers CD81, CD9, and Tsg101, while the negative marker Calnexin was not detected in AMSC‐exo. (D–G) RT‐qPCR analysis of Hif‐1α, α‐SMA, Collagen I, and TGF‐β mRNA levels in HK2 cells (*n* = 4). (H, I, K) Representative Western blots and quantification of Hif‐1α and α‐SMA protein expression in mouse kidneys (*n* = 3). (J) HE, Masson's trichrome, and α‐SMA immunohistochemistry (IHC) demonstrate tubulointerstitial changes (scale bar: 100 μm). These data are presented as the means ± standard deviation (SD) of three independent experiments, with statistical significance indicated (*p < 0.05, **p < 0.01, ***p < 0.001; one‐way ANOVA).

### 3.2 AMSC‐Exo Ameliorates Renal Hypoxia and Fibrosis in a UUO Model

To probe the therapeutic potential of AMSC‐exo in kidney injury, we established a UUO mouse model. Western blotting analysis of kidney tissues revealed a significant upregulation of HIF‐1α (hypoxia marker) and α‐SMA (fibrosis marker) in the UUO group compared to the sham (Figure 1H,I,K). H&E and Masson's staining of kidney sections depicted normal architecture in the sham group, while the UUO group exhibited tubular dilation, tubular epithelial cell apoptosis, hypotrophy, mild and scattered inflammatory cell infiltration, and limited fibrosis at 7 days post‐injury (Figure 1J). Notably, AMSC‐exo administration in the UUO group resulted in a marked reduction in these pathological changes, including decreased tubular dilation, remission of tubular epithelial cell damage, and significant improvement in fibrosis. Additionally, minimal inflammatory cell infiltration was observed. α‐SMA IHC confirmed its increased expression in the UUO group and efficient suppression by AMSC‐exo treatment (Figure 1J). In conclusion, AMSC‐exo treatment significantly attenuated renal injury in UUO mice, likely via improved oxygenation, reduced fibrosis, and mitigated inflammation.

### 3.3 AMSC‐Exo Ameliorates Fibrosis and Enhances HK2 Proliferation and Migration In Vitro

Echoing the in vivo findings, HK2 cells under hypoxia displayed increased HIF‐1α and α‐SMA protein levels compared to the control (Figure 2A,C,D). AMSC‐exo treatment partially reversed this, reducing HIF‐1α and α‐SMA expression, suggesting mitigation of hypoxia and fibrosis. Consistently, the protein expression levels of Collagen I and TGF‐β1 were markedly increased under hypoxic conditions and were significantly reduced following AMSC‐exo treatment (Figure 2B,G,H). We further evaluated the effects of AMSC‐exo on HK2 cell migration and proliferation. Transwell assays revealed that hypoxia treatment significantly decreased migration by 45.77% compared to the control group. Notably, AMSC‐exo administration partially restored migration, resulting in a migration decrease of only 23.61% compared to control (Figure 2E,I). An EdU proliferation assay demonstrated that hypoxia treatment substantially suppressed HK2 cell proliferation (control: 61.20% ± 2.90%; hypoxia: 12.02% ± 5.78%; hypoxia + AMSC‐exo: 38.99% ± 1%). Importantly, AMSC‐exo administration significantly restored HK2 cell proliferation inhibited by hypoxia (Figure 2F,J).

**FIGURE 2:**
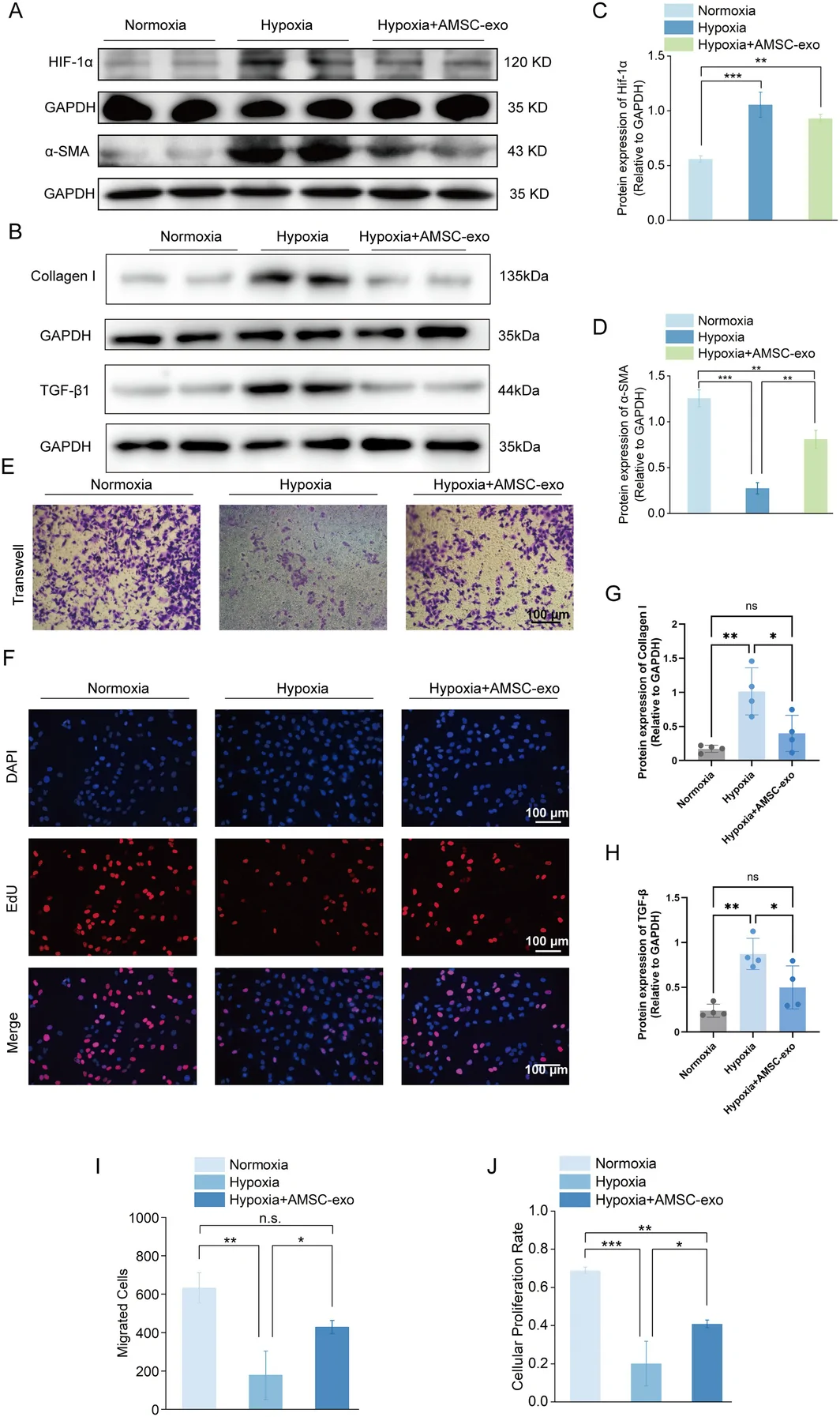
AMSC‐exo modulates HK2 proliferation and migration in vitro. (A) Representative Western blot images of Hif‐1α and α‐SMA protein expression in HK2 cells under different treatments. (B) Representative Western blot images of Collagen I and TGF‐β1 protein expression in HK2 cells under different treatments. (C, D) Quantification of Hif‐1α and α‐SMA protein levels, respectively (*n* = 4). (G, H) Quantification of Collagen I and TGF‐β1 protein levels, respectively (*n* = 4). (E, I) Representative images and quantification of HK2 cell migration assessed by Transwell assay (scale bar: 100 μm) (*n* = 3). (F, J) Representative images and quantification of HK2 cell proliferation assessed by EdU assay (scale bar: 100 μm) (*n* = 3). These data are presented as the means ± standard deviation (SD) of three independent experiments, with statistical significance indicated (*p < 0.05, **p < 0.01, ***p < 0.001; one‐way ANOVA).

### 3.4 miR‐26a‐5p Identifies as a Candidate Regulator in AMSC‐Exo

To decipher the mechanism underpinning the anti‐fibrotic effects of AMSC‐exo, we utilized miRNA sequencing to identify enriched miRNAs within the exosomes. This analysis unveiled the five most abundant miRNAs: miR‐26a‐5p, miR‐150‐5p, miR‐1301‐3p, miR‐193a‐3p and miR‐33a‐3p, collectively constituting nearly 48% of the total miRNA population (Figure 3A). Given its prevalence in AMSC‐exo and supporting literature, we further probed the specific role of exosomal miR‐26a‐5p in renal tubular epithelial cells. Through a combined analysis of online databases such as miWalk and TargetScan, we identified Pfkfb3 as a potential target gene for miR‐26a‐5p (Figure 3B,C). We generated miR‐26a‐5p inhibitor‐transfected AMSCs (miR‐26a‐5pIN‐AMSC) and NC‐transfected AMSCs (NC‐AMSC) to isolate exosomes with miR‐26a‐5p inhibitor (miR‐26a‐5pIN‐AMSC‐exo) and control exosomes (NC‐Exo). As anticipated, miR‐26a‐5p expression was suppressed in miR‐26a‐5pIN‐AMSC (Figure 3D). Dual‐luciferase reporter assay confirmed Pfkfb3 as a target of miR‐26a‐5p (Figure 3E).

**FIGURE 3:**
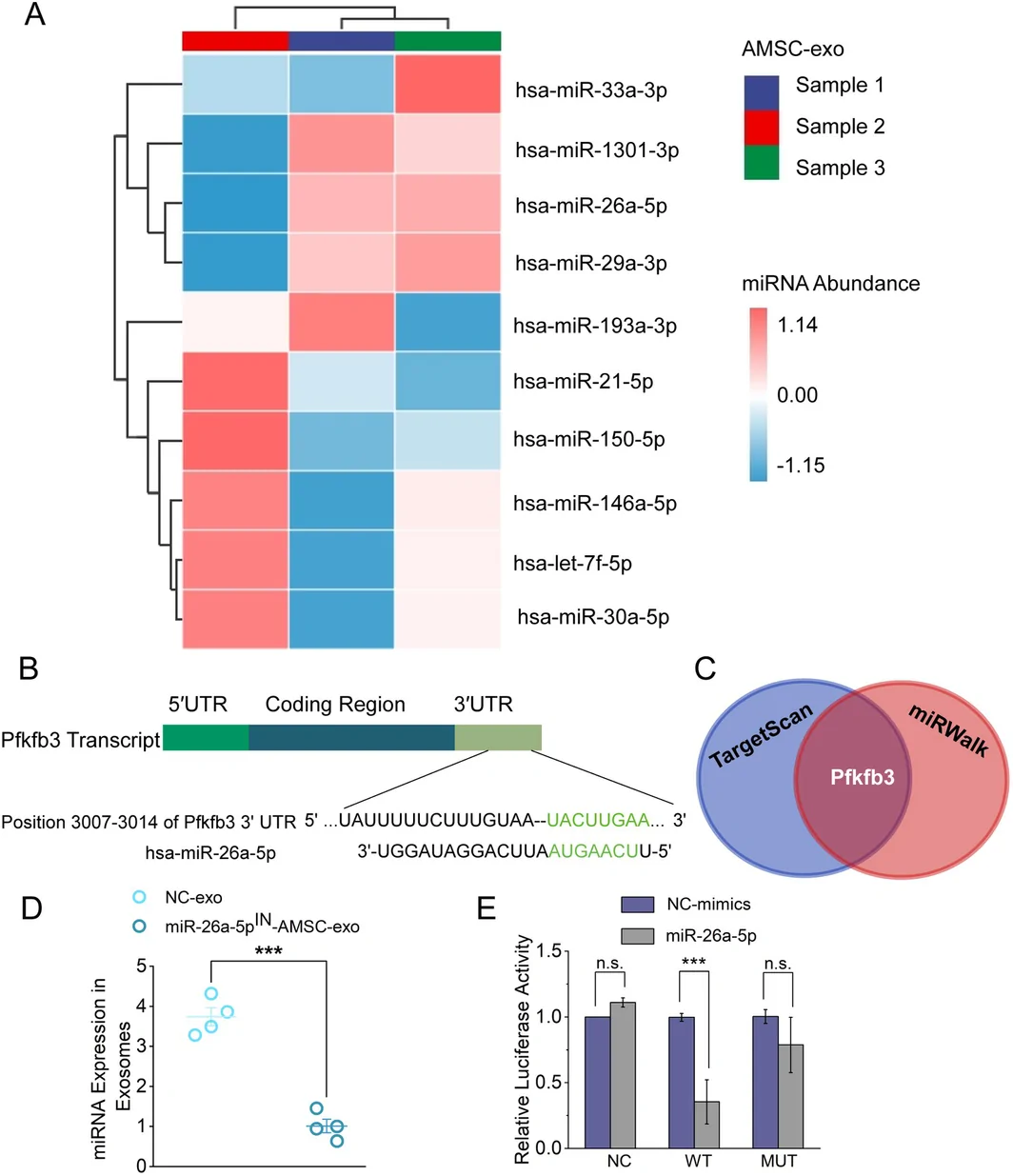
miR‐26a‐5p enrichment in AMSC‐exo and targeting of Pfkfb3. (A) Heatmap shows the top ten most abundant miRNAs in AMSC‐exo identified by miRNA sequencing. (B) Schematic diagram depicts the predicted miR‐26a‐5p binding site in the Pfkfb3 3′UTR. (C) Venn diagram highlights Pfkfb3 as a shared target gene predicted by TargetScan and miRWalk. (D) RT‐qPCR confirms miR‐26a‐5p expression in AMSC‐exo after transfection with miR‐26a‐5p inhibitor or NC vector (*n* = 3). (E) Dual‐luciferase reporter assay validates the conserved miR‐26a‐5p binding site in the Pfkfb3 3′UTR (*n* = 3). *p < 0.05, **p < 0.01, ***p < 0.001 (one‐way ANOVA).

When HK2 cells subjected to hypoxia and UUO mice were treated with AMSC‐exo or AMSC‐miR‐26a‐5pIN‐Exo (AMSC‐26a‐5pIN‐Exo), the knockdown of miR‐26a‐5p blocked the ability of AMSC‐exo to reduce Pfkfb3 levels (Figure 4A,B). Notably, AMSC‐26a‐5pIN‐Exo reversed the increase in cellular migration and proliferation induced by AMSC‐exo treatment following hypoxia (Figure 4C,D). These findings collectively demonstrate that AMSC‐exo enhanced HK2 viability by downregulating Pfkfb3 through miR‐26a‐5p. Moreover, H&E and Masson staining revealed that the knockdown of miR‐26a‐5p exacerbated kidney damage and fibrosis, accompanied by the upregulation of Pfkfb3 (Figure 4E). These findings collectively demonstrate that AMSC‐exo protected against renal fibrosis by downregulating Pfkfb3 through miR‐26a‐5p.

**FIGURE 4:**
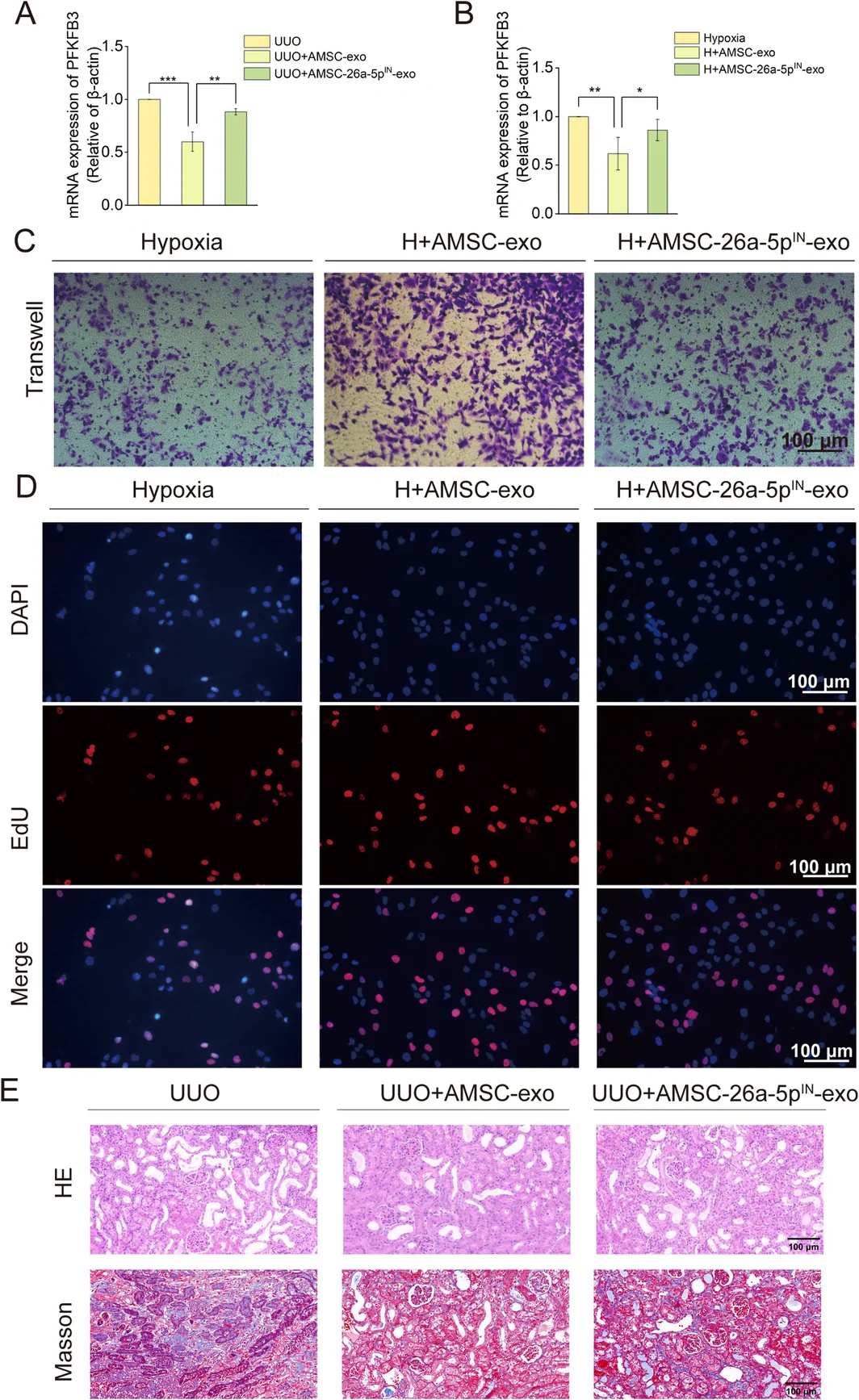
miR‐26a‐5p inhibition blocks AMSC‐exo's protective effects. (A, B) RT‐qPCR analysis of Pfkfb3 mRNA levels in HK2 cells treated with NC‐exo, miR‐26a‐5p inhibitor (miR‐26a‐5pIN)‐exo, or AMSC‐exo (*n* = 4), both in vivo and in vitro. *p < 0.05, **p < 0.01, ***p < 0.001 (one‐way ANOVA). (C, D) Representative transwell migration and EdU proliferation assays show the impacts of miR‐26a‐5pIN‐exo or AMSC‐exo on hypoxic HK2 cells. Scale bars: 100 μm. (E) Representative HE and Masson's trichrome staining of renal tissues from UUO mice treated with miR‐26a‐5pIN‐exo or AMSC‐exo. Scale bars: 100 μm.

### 3.5 AMSC‐Exo Attenuates Renal Fibrosis by Suppressing Glycolysis in HK2 Cells Targeting Pfkfb3

Tubular epithelial cells, pivotal for renal function and energy‐intensive, are disrupted by hypoxia, instigating a shift toward the less efficient energy production pathway, aerobic glycolysis. Our investigation identified Pfkfb3, a key glycolytic enzyme, as a potential target. Notably, AMSC‐exo, enriched in miR‐26a‐5p, can diminish Pfkfb3 expression, suggesting a potential mechanism by which AMSC‐exo might interfere with glycolysis.

To ascertain the impact of Pfkfb3 on glycolysis, we performed RNA sequencing, comparing gene expression profiles between the UUO and sham groups. The volcano plot depicted 3377 upregulated and 2646 downregulated genes (Figure 5A). Subsequently, reactome enrichment analysis was employed to identify enriched metabolic pathways among the differentially expressed genes (Figure 5B). Finally, we identified all genes involved in the identified metabolic pathway and generated a heatmap for visualization (Figure 5C). These findings imply that AMSC‐exo may regulate glycolysis via the miR‐26a‐5p/Pfkfb3 axis, potentially offering a novel therapeutic strategy for renal fibrosis amelioration.

**FIGURE 5:**
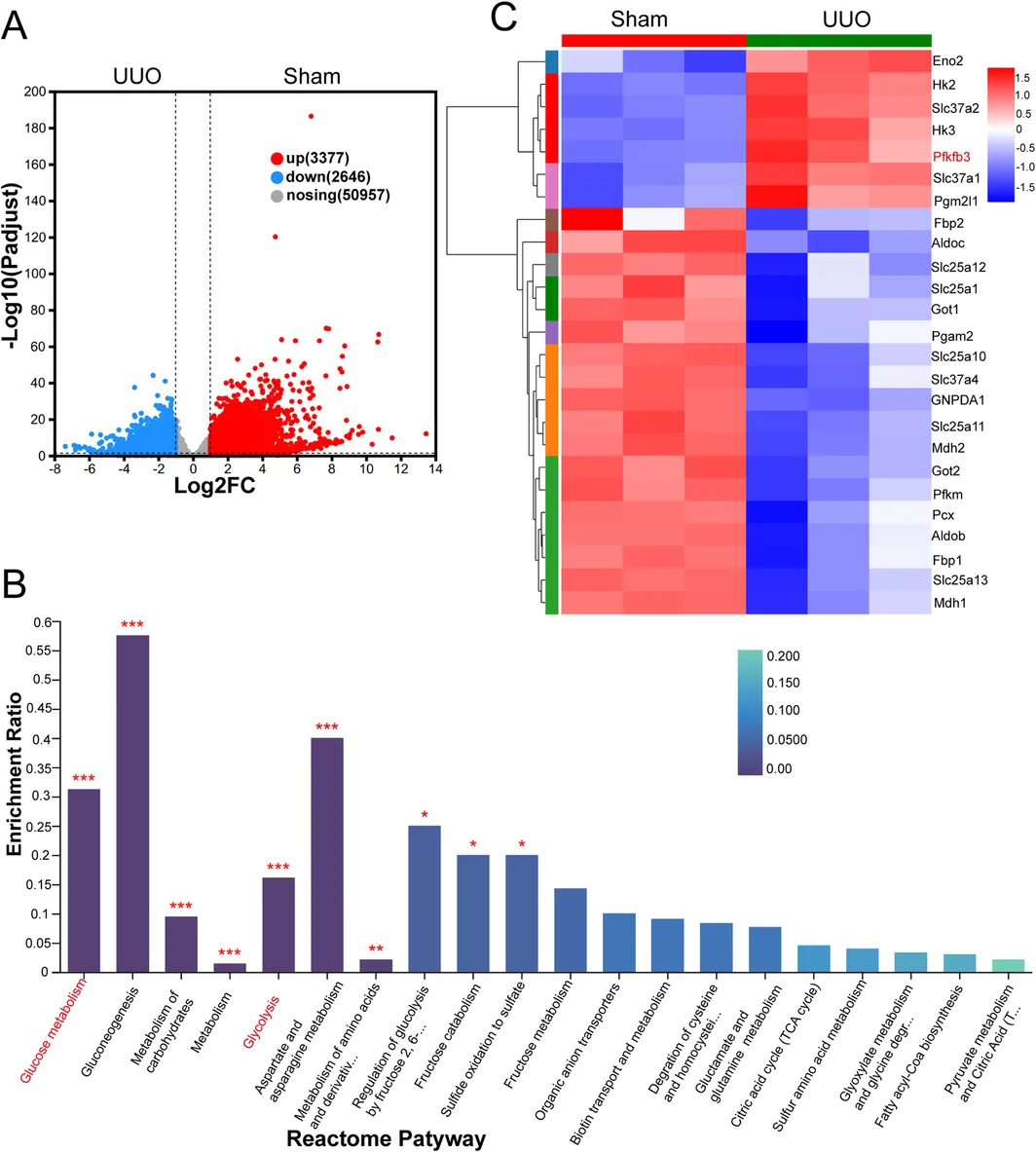
Pfkfb3 enrichment and metabolic pathway dysregulation in UUO kidneys. (A) Volcano plot highlights differentially expressed genes between sham and UUO groups. Each dot represents a gene, with colour indicating fold change. (B) Reactome pathway enrichment analysis identifies pathways significantly associated with the differentially expressed genes, potentially pointing toward glucose metabolism pathways involved in renal injury and repair. (C) Heatmap depicts the expression levels of key differentially expressed genes, particularly those involved in metabolic pathways, potentially contributing to UUO‐induced renal injury. *p < 0.05, **p < 0.01, ***p < 0.001 (one‐way ANOVA).

To evaluate the impact of AMSC‐exo on glycolysis, we analysed the expression of key glycolytic enzymes in hypoxia‐treated HK2 cells and UUO mice alongside either NC‐Exo or miR‐26a‐5pIN‐Exo. RT‐qPCR revealed that miR‐26a‐5p knockdown regulated the expression of several key checkpoint enzymes, such as hexoskinase 2 (HK2), Phosphofructokinase, muscle type (PFKM), and lactate dehydrogenase A (LDHA). Conversely, HK2 cells and UUO mice treated with AMSC‐exo exhibited a marked downregulation of these enzymes (Figure 6A–F).

**FIGURE 6:**
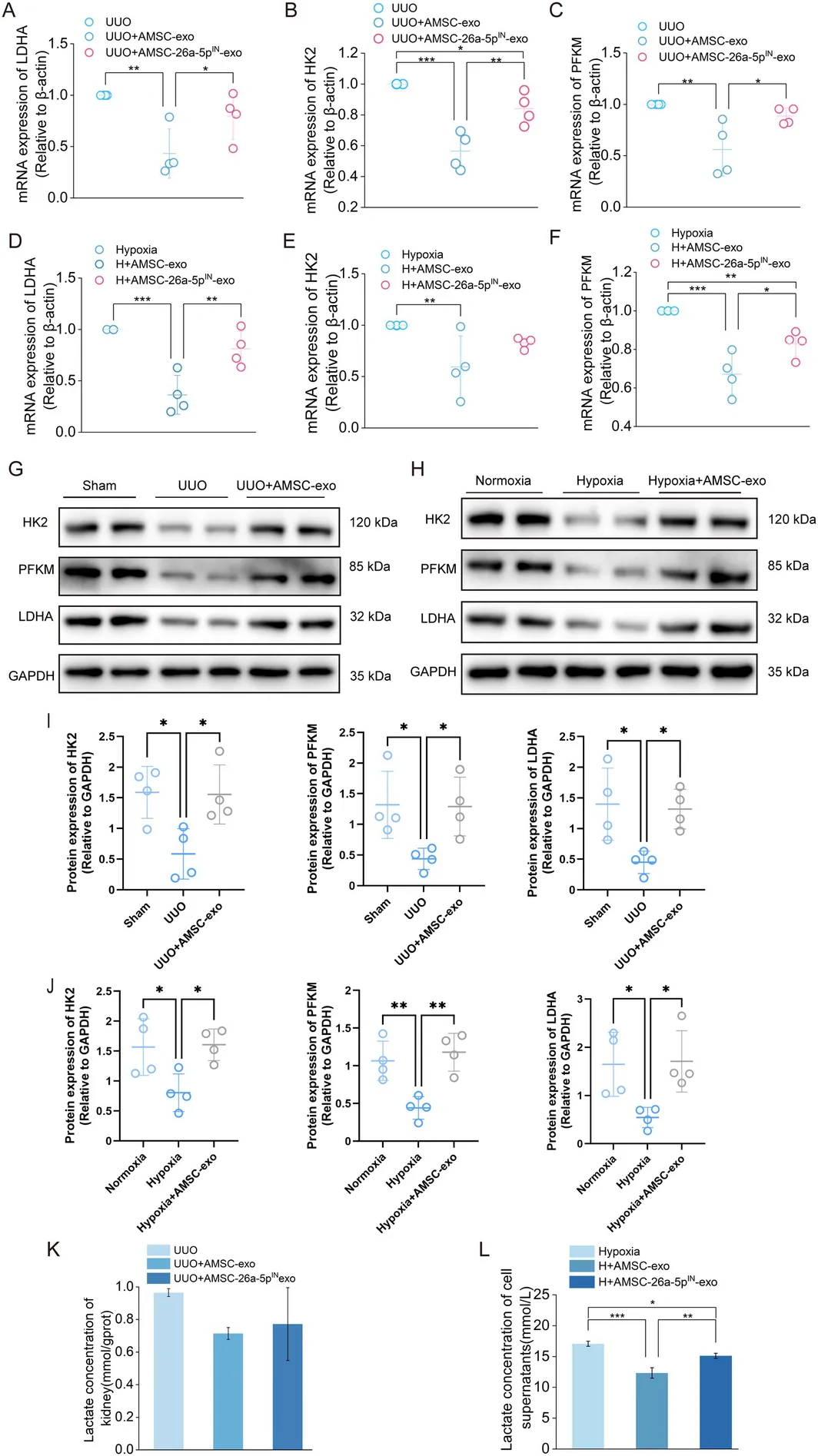
AMSC‐exo suppresses glycolysis via miR‐26a‐5p‐mediated Pfkfb3 inhibition. (A–F) RT‐qPCR analysis demonstrates the mRNA expression levels of key glycolytic enzymes (LDHA, HK2, PFKM) in HK2 cells under hypoxia and in UUO mice treated with miR‐26a‐5p inhibitor‐AMSC‐exo or AMSC‐exo (*n* = 4). *p < 0.05, **p < 0.01, ***p < 0.001 (one‐way ANOVA). (G, H) Representative Western blot images of HK2, PFKM, and LDHA protein expression in UUO mouse kidneys and hypoxia‐treated HK2 cells. (I, J) Quantification of HK2, PFKM, and LDHA protein expression levels (*n* = 4). (K, L) Quantification of lactate concentration, a marker of glycolysis, in vitro and in vivo (*n* = 3). *p < 0.05, **p < 0.01, ***p < 0.001 (one‐way ANOVA).

Consistent with the mRNA findings, Western blot analysis further demonstrated that the protein expression levels of HK2, PFKM, and LDHA were significantly increased in the hypoxia and UUO groups and were markedly reduced following AMSC‐exo treatment (Figure 6G–J).

Subsequently, we assessed the lactate concentration in HK2 cells and kidney tissues among each group (Figure 6K,L). The results indicated relatively higher lactate concentrations in the miR‐26a‐5pIN‐Exo‐treated group compared to the hypoxia and UUO groups. Conversely, lactate production was significantly downregulated in the AMSC‐exo treatment. Collectively, the data support the notion that AMSC‐exo serves as a potent regulator of glycolysis in HK2 cells via miR‐26a‐5p targeting Pfkfb3, potentially contributing to the attenuation of renal fibrosis.

### 3.6 Potential Role of Pfkfb3 in Ferroptosis and Renal Fibrosis

Pfkfb3, a critical glycolytic enzyme, has been implicated in regulating ferroptosis, an iron‐dependent cell death form. Research suggests a correlation between ferroptosis and mitochondrial health, which can be compromised by energy metabolism imbalances. Transcriptome analysis revealed a significant upregulation of Pfkfb3 in the UUO group. Pfkfb3 encodes a key enzyme involved in cellular metabolism regulation, suggesting that altered metabolism might be associated with ferroptosis in kidney injury. Based on this, we hypothesize that Pfkfb3 might regulate ferroptosis in HK2 cells, potentially influencing renal fibrosis progression.

Further investigation into the potential role of ferroptosis revealed mitochondrial damage in the UUO and miR‐26a‐5pIN‐Exo groups through TEM. In contrast, AMSC‐exo appeared to protect mitochondria (Figure 7A). This confirms the destruction of mitochondria in the UUO and miR‐26a‐5pIN‐Exo groups, with AMSC‐exo mitigating this destruction. Moreover, the knockdown of miR‐26a‐5p, resulting in elevated Pfkfb3 levels, precipitated an intensification of the ferroptosis process.

**FIGURE 7:**
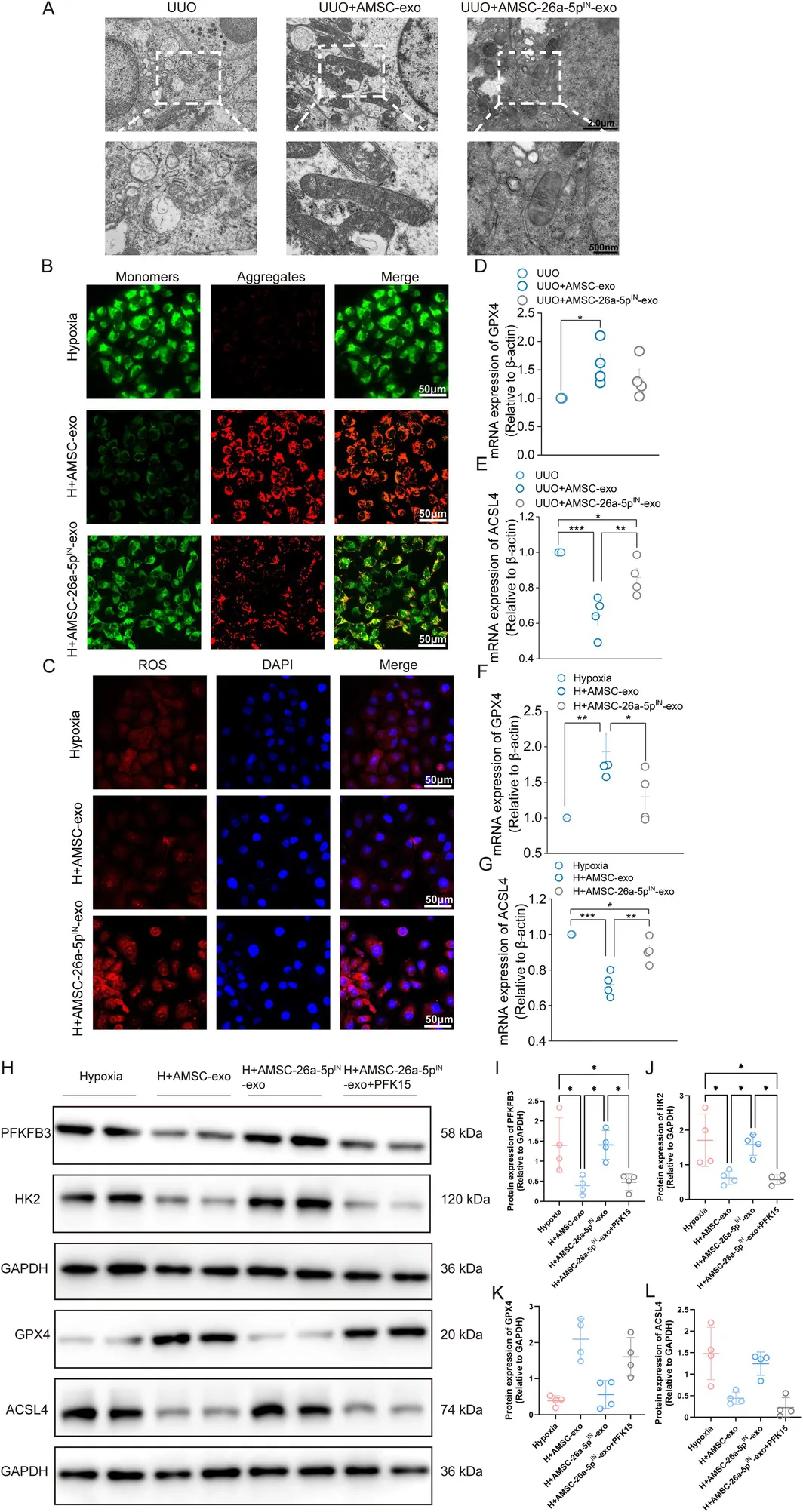
miR‐26a‐5p inhibition exacerbates and Pfkfb3 inhibition reverses these effects. (A) TEM reveals changes in mitochondrial morphology and number in kidney tissues. Scale bars: 2 μm and 500 nm. (B) Representative images demonstrate alterations in mitochondrial membrane potential. Scale bars: 50 μm. (C) Representative images depict mitochondrial ROS production. Scale bars: 50 μm. (D‐G) RT‐qPCR analysis confirms mRNA expression levels of key ferroptosis regulators (GPX4, ACSL4) in HK2 cells under hypoxia and UUO mice treated with miR‐26a‐5p inhibitor‐AMSC‐exo or AMSC‐exo (*n* = 4). (H) Western blot analysis of Pfkfb3, HK2, GPX4, and ACSL4 protein expression in HK2 cells under different treatment conditions, including hypoxia, AMSC‐exo, miR‐26a‐5p inhibitor, and PFK15. (I–L) Quantitative analysis of protein expression levels corresponding to (H), demonstrating that Pfkfb3 inhibition by PFK15 reverses the effects of miR‐26a‐5p knockdown on glycolysis‐ and ferroptosis‐related markers (*n* = 4). *p < 0.05, **p < 0.01, ***p < 0.001 (one‐way ANOVA).

JC‐1 staining confirmed increased mitochondrial dysfunction in the hypoxia and miR‐26a‐5pIN‐Exo groups (Figure 7B). Furthermore, the analysis of mitochondrial superoxide production, a major type of ROS generated in the mitochondria, showed elevated levels in the hypoxia and miR‐26a‐5pIN‐Exo groups compared to the control (Figure 7C).

To further explore the connection between Pfkfb3 and ferroptosis, we performed RT‐qPCR analysis to examine the expression of key ferroptosis regulators. The results revealed that compared to the AMSC‐exo treatment group, GPX4, an enzyme known to protect against ferroptosis, was significantly downregulated in both the hypoxia and UUO groups. Conversely, the expression of ACSL4, an enzyme involved in ferroptosis execution, was upregulated in these groups. Moreover, the knockdown of miR‐26a‐5p exacerbated this change. These findings suggest a crucial link between ferroptosis and Pfkfb3 activity (Figure 7D–G).

Importantly, to determine whether Pfkfb3 is a functional mediator downstream of miR‐26a‐5p, we performed a rescue experiment using PFK15, a specific Pfkfb3 inhibitor. Western blot analysis demonstrated that inhibition of Pfkfb3 markedly reversed the effects induced by miR‐26a‐5p knockdown, as evidenced by reduced expression of Pfkfb3 and HK2, restoration of GPX4 levels, and suppression of ACSL4 expression (Figure 7H–K). These findings indicate that Pfkfb3 inhibition attenuates both glycolytic activation and ferroptosis triggered by miR‐26a‐5p deficiency.

Together, these results demonstrate that Pfkfb3 acts as a key downstream functional mediator of miR‐26a‐5p, linking glycolytic reprogramming to ferroptosis in tubular epithelial cells.

## 4 Discussion

This study probes the therapeutic potential of AMSC‐exo in mitigating renal injury. Bioinformatics analysis identified miR‐26a‐5p as a significant constituent of AMSC‐exo, with further validation affirming its interaction with Pfkfb3, a key glycolysis and ferroptosis regulator in TECs. Importantly, our data demonstrate that AMSC‐exo attenuate renal fibrosis by modulating the miR‐26a‐5p/Pfkfb3 axis, thereby regulating glycolysis and ferroptosis in tubular epithelial cells. These findings suggest that miR‐26a‐5p‐enriched AMSC‐exo can promote HK2 cell proliferation and migration, potentially offering a novel approach for renal repair and prevention of CKD progression (Figure 8).

**FIGURE 8:**
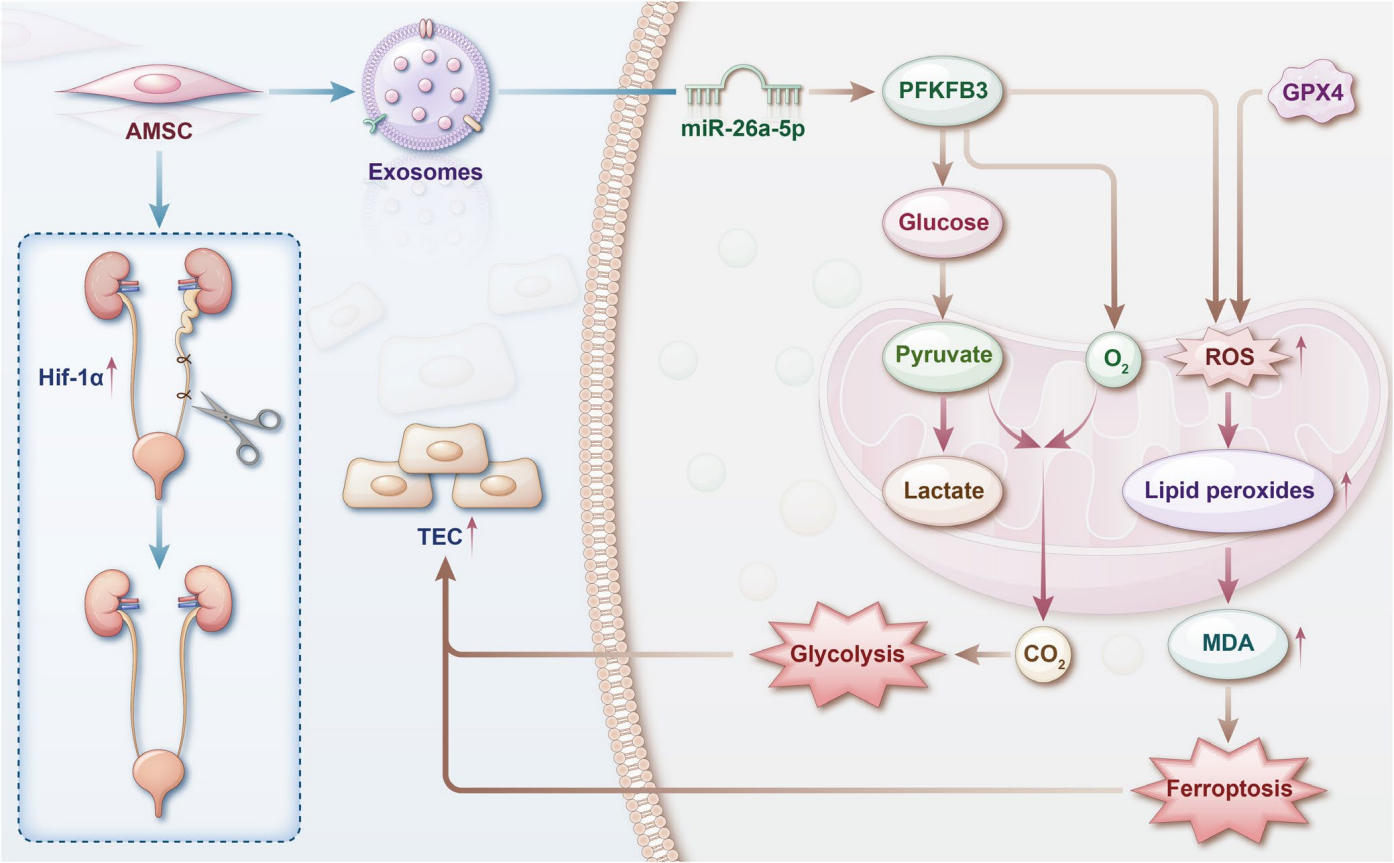
Schematic illustration of the protective mechanism of AMSC‐derived exosomes in renal fibrosis. AMSC‐exo deliver miR‐26a‐5p into tubular epithelial cells, where miR‐26a‐5p directly targets and suppresses the expression of Pfkfb3. Downregulation of Pfkfb3 inhibits glycolytic reprogramming by reducing the expression of key glycolytic enzymes, including HK2, PFKM, and LDHA, leading to decreased lactate production. Meanwhile, suppression of Pfkfb3 also attenuates ferroptosis, as evidenced by increased GPX4 expression and decreased ACSL4 expression, along with improved mitochondrial function and reduced ROS accumulation. Through coordinated inhibition of glycolysis and ferroptosis, AMSC‐exo ultimately alleviates tubular epithelial cell injury and renal interstitial fibrosis.

Renal fibrosis, a hallmark of CKD and a major prognostic factor, can potentially be combated using MSC‐derived exosomes [20]. To focus on early fibrogenic events relevant to our mechanistic investigation, a 7‐day UUO model was employed. At this stage, hallmark features of renal fibrosis, including activation of myofibroblasts (e.g., increased α‐SMA expression) and initiation of extracellular matrix deposition, are already present, whereas the overall tissue architecture remains relatively preserved. Importantly, this time point enables the evaluation of early molecular and metabolic alterations in tubular epithelial cells, which are central to our study. In contrast, longer durations (e.g., 14 days) are typically associated with advanced fibrosis and extensive tissue damage, which may obscure early regulatory mechanisms [21]. Consistent with previous studies, MSC‐derived exosomes exhibit anti‐fibrotic potential [14, 22] our findings further extend this concept by identifying a specific miRNA‐mediated metabolic regulatory mechanism.

Mesenchymal stem cells have been widely studied for their regenerative potential, largely attributed to their paracrine effects mediated by extracellular vesicles. Emerging evidence underscores the therapeutic promise of MSC‐derived extracellular vesicles, notably exosomes, in CKD management [23, 24]. In line with these observations, our study demonstrates that AMSC‐exo significantly improves TEC survival and function under hypoxic conditions. Notably, we identified miR‐26a‐5p as a highly enriched miRNA within AMSC‐exo and demonstrated that inhibition of miR‐26a‐5p markedly weakened the protective effects of AMSC‐exo, confirming its functional importance. These findings establish miR‐26a‐5p as a critical mediator of AMSC‐exo‐driven renal protection.

A key finding of this study is the identification of Pfkfb3 as a downstream target of miR‐26a‐5p. Pfkfb3 plays a central role in glycolytic regulation, and metabolic reprogramming toward aerobic glycolysis has been increasingly implicated in renal fibrosis [25]. TECs, requiring substantial oxygen, are particularly susceptible to hypoxia. In response to hypoxia and other stressors, TECs transition from fatty acid degradation to aerobic glycolysis for energy production [26, 27]. Our data demonstrate that hypoxia and UUO induce upregulation of key glycolytic enzymes (HK2, PFKM, and LDHA) at both mRNA and protein levels, accompanied by increased lactate production, indicating enhanced glycolysis. Importantly, AMSC‐exo treatment significantly reversed these changes, supporting the notion that AMSC‐exo suppress glycolytic reprogramming in TECs via miR‐26a‐5p‐mediated inhibition of Pfkfb3. Our findings further suggest that Pfkfb3 serves as a functional link between metabolic reprogramming and ferroptosis regulation in TECs, consistent with emerging evidence that enhanced glycolysis can sensitize cells to ferroptotic stress.

Energy metabolism disorders, typified by ROS overproduction and frequently accompanied by mitochondrial dysfunction, can instigate ferroptosis [28]. Ferroptosis is a regulated form of cell death driven by iron‐dependent lipid peroxidation and oxidative stress [29, 30]. Research has extensively probed the role of ferroptosis in both acute kidney injury and renal cell carcinoma. Emerging evidence also indicates a connection between ferroptosis and renal fibrosis [31, 32]. Our study further disclosed that Pfkfb3, regulated by miR‐26a‐5p, can modulate ferroptosis [33]. We noted that UUO mice induced morphological destruction and mitochondrial reduction in the kidneys. These deleterious effects were counteracted by AMSC‐exo treatment. Conversely, miR‐26a‐5p knockdown elevated Pfkfb3 expression, leading to alterations in key ferroptosis enzyme expression and increased mitochondrial ROS production in TECs. This ultimately exacerbated the degree of cell necrosis. Importantly, our rescue experiments further support a causal role of Pfkfb3 in mediating this process. These results suggest a mechanistic link between glycolysis and ferroptosis mediated by the miR‐26a‐5p/Pfkfb3 axis.

Taken together, our findings support a model in which AMSC‐exo deliver miR‐26a‐5p to TECs, leading to suppression of Pfkfb3 expression, attenuation of glycolytic reprogramming, and inhibition of ferroptosis, ultimately alleviating renal fibrosis. This integrated regulatory mechanism highlights the interplay between cellular metabolism and cell death pathways in renal injury.

In conclusion, AMSC‐exo attenuates renal fibrosis by suppressing glycolysis and ferroptosis in TECs through the miR‐26a‐5p/Pfkfb3 axis. This study provides novel mechanistic insight into the therapeutic potential of AMSC‐exo and identifies a promising target for the treatment of renal fibrosis.

## Author Contributions

**Chi Zhang:** writing – original draft, funding acquisition. **Rui Ge:** methodology. **Min Liu:** data curation. **Jianhuai Huang:** investigation. **Jingyu Cai:** data curation. **Tao Sun:** software. **Sheng Ge:** visualization. **Qiuchen Li:** software. **Yuanyuan Zhang:** project administration, writing – review and editing.

## Funding

This study was supported by research project funding of Jiangsu Provincial Health Commission (Nos. MQ2024026 and Ym2023037); Development Fund of Suqian Sci&Tech Program (Nos. K202512 and K202313); Jiangsu Key Laboratory of Immunity and Metabolism (No. XZSYSKF2022041); Development Fund of Affiliated Hospital of Xuzhou Medical University (No. XYFY202236); Jiangsu Maternal and Child Health Program (Grant No. FYX202333); Jiangsu Provincial Young Scientific and Technological Talents Support Program (No. JSTJ‐2024‐656); Suzhou Sci&Tech Program Medical Application Basic Research (No. SKYD2023093).

## Ethics Statement

TargetScan and miRWalk are freely accessible online databases providing miRNA target prediction data for research purposes. Animal experiments followed a pre‐approved protocol by the Institutional Animal Care and Use Committee of Xuzhou Medical University (permit number: L20210226432).

## Consent

The authors have nothing to report.

## Conflicts of Interest

The authors declare no conflicts of interest.

## Data Availability

The data that support the findings of this study are available from the corresponding author upon reasonable request.
